# Effect of active fraction isolated from the leaf extract of *Dregea volubilis* [Linn.] Benth. on plasma glucose concentration and lipid profile in streptozotocin-induced diabetic rats

**DOI:** 10.1186/2193-1801-2-394

**Published:** 2013-08-21

**Authors:** Venkatesan Natarajan, Anton Smith Arul Gnana Dhas

**Affiliations:** Department of Pharmacy, Annamalai University, Annamalai Nagar, Tamil nadu 608 002 India

**Keywords:** Blood glucose, Hexadecenoic acid, *Dregea volubilis* [Linn.] Benth, Lipid profile

## Abstract

**Objective:**

The objective of the present study was to evaluate the effect of active fraction from *Dregea volubilis* [Linn.] Benth leaves on serum glucose and lipid profile in normal and diabetic rats.

**Materials and methods:**

Diabetes was induced by streptozotocin in wistar rats. Petroleum ether, ethyl acetate, and ethanol extracts of *Dregea volubilis* [Linn.] Benth leaves were administered orally at a dose of 200 mg/kg, p.o. Metformin was used as standard Anti-diabetic drug (50 mg/kg, p.o). The extract showing for higher Anti-diabetic activity was subjected to column chromatography that led to isolation of an active fraction, which was given trivial name Dv-1. Dv-1 (100 mg/kg, p.o.) was studied for its hypoglycemic and hypolipidemic potential.

**Results:**

Ethanol extract was found to lower the Fasting blood glucose (FBG) level significantly (*p* < 0.05) in diabetic rats. Dv-1 caused a significant (*p* < 0.05) reduction in FBG level. Additionally it also caused reduction in cholesterol, triglyceride levels and improvement in the HDL level in diabetic rats.

**Conclusion:**

Reduction in the FBG, cholesterol, triglyceride levels and improvement in the HDL by Dv-1 indicates that Dv-1 has Anti-diabetic activity along with anti hyperlipidemic efficacy and provides a scientific rationale for the use as an Anti-diabetic agent.

## Introduction

Diabetes is a complex and a multivarious group of disorders that disturbs the metabolism of carbohydrates, fat and protein (Kahn et al. 1991) characterized by increased fasting and postprandial blood sugar levels. Diabetes mellitus is classified into two major subtypes: type I (insulin dependent diabetes mellitus, IDDM) and type II (non-insulin dependent diabetes mellitus, NIDDM). IDDM or juvenile-onset diabetes results from a cellular mediated autoimmune destruction of the β-cells of the pancreas (Aikinson et al. 1994). However, NIDDM or adult-onset diabetes results from the development of insulin resistance and the affected individuals usually have insulin deficiency (Takeshi et al. 2002). Type II diabetes is the most common form of diabetes constituting 90% of the diabetic population. In 1995 it was estimated that around 135 million people were affected from this condition and it was expected to affect 300 million by the year 2025 (King et al. 1998). Management of diabetes without any side effect is still a challenge to the medical community. Several drugs such as biguanides, sulfonylurea and thiazolidenediones are presently available to treat the diabetes mellitus (De-Fronzo et al. 1997). The use of these drugs is restricted by their pharmacokinetic properties, secondary failure rates and accompanying side effects (Donath et al. 2006). Thus searching for a new class of compounds is essential to overcome diabetic problems ultimately leading to continuous search for alternative drugs (Hansotia et al. 2005). The medicinal plants may provide the useful source of new oral hypoglycemic compounds for the development of pharmaceutical entities or as dietary adjunct to existing therapies (Pepato et al. 2005). Furthermore, after the recommendation made by WHO on diabetes mellitus, investigation on hypoglycemic agents from medicinal plants have become more important. The ethnobotanical information reports state that about 800 plants may possess Anti-diabetic potential (Grover et al. 2004). Recently the medicinal values of various plant extracts have been studied by many scientists in the field of diabetic research (Jain et al. 2010). The rational design of novel drugs from traditional medicine offers new prospects in modern healthcare. More than 800 plants are used as traditional remedies for the treatment of diabetes throughout the world (Kavishankar et al. 2011). *Dregea volubilis* [Linn.] Benth belongs to family Asclepiadaceae is widely used in Indian traditional medicines and the leaf paste is used to treat rheumatic pain, cough, fever and severe cold (Rajadurai et al. 2009). Leaf paste is taken along with pepper to treat dyspepsia (Pandikumar et al. 2007); bark paste mixed with hot milk is used internally for treating urinary infections (Silija et al. 2008). The plant is being used very specifically in the indigenous systems of medicine such as Ayurveda, Siddha and Unani. The present study was planned to compare the blood glucose-lowering activity of different extracts/fractions of *Dregea volubilis* in order to isolate the active principle responsible for the Anti-diabetic activity of the plant. Further study was carried out to evaluate the Anti-diabetic and hypolipidemic effects of active principle (Dv-1) from *Dregea volubilis* leaf extract on normal and diabetic rats.

## Methods

### Drugs and chemicals

Streptozotocin (STZ) was purchased from Sigma aldrich, Bangalore, India. Standard Anti-diabetic drug Metformin was obtained from Actavis pharmaceutical, Chennai, India. Analytical grade of chemicals, including various organic solvents (petroleum ether, ethyl acetate, chloroform, ethanol and methanol) from S.D. Fine Chemicals, India, were used for the extraction and the phytochemical study of the constituents.

### Preparation of different plant extracts

*Dregea volubilis* leaves were collected from the forest of kalakatu, Tirunelveli District, India. Taxonomic identification was made from botanical survey of medicinal plants, Siddha Unit, Government of India, Palayamkottai authenticated by Chelladurai Botanist. A voucher specimen No (CCRAS-167/2011). Fresh plant leaves were shade dried at room temperature, ground into fine powder and stored in airtight containers. Then extracted (amount 500 g) with solvents of increasing polarity such as petroleum ether, ethyl acetate, and ethanol, for 72 hours with each solvent, by continuous hot extraction using the soxhlet apparatus at a temperature of 60°C. The extracts were concentrated under reduced pressure using a rotary evaporator to constant weight. The extracts were collected and preserved in a desiccator until used for further studies. The percentage yields were 10.81% in petroleum ether, 11.36% in ethyl acetate and 12.13% in ethanol.

### Acute toxicity study

Acute toxicity study was performed according to OECD- 423 guidelines. Albino rats (n = 6) of either sex selected by random sampling technique were employed in this study. The animals were fasted for 4 h with free access to water only. The various extract of *Dregea volubilis* suspended in normal saline: tween 80 (95:5) which was administered orally at a dose of 5 mg/kg initially and mortality was observed for 3 days. The mortality was not observed, the procedure was then repeated with higher doses such as 50, 300, 1000, and 2000 mg/kg. The mortality was observed in 5/6 or 6/6 animals when administered with the dose of 2000 mg/kg and then the dose administered was considered as toxic dose. However, the mortality was observed in less than four rats, out of six animals then the same dose was repeated again to confirm the toxic effect.

### Animals

Male wistar rats each weighing 180–220 g was obtained from Raja Muthiah Medical College and Hospital (RMMCH) in Annamalai University at Chidambaram, Tamil nadu, India. The guidelines of the Committee for the purpose of control and supervision of experiments on animals (CPCSEA) of the Government of India were followed and prior permission was granted from the Institutional animal ethics committee (NO.842/CPCSEA). Rodent laboratory chow was access and water *ad libitum*, and rats were maintained on a 12 hour light/dark cycle in a temperature regulated room (20–25°C) during the experimental procedures.

### Induction of diabetes

The fasted rats (Sriplang et al. 2007) were injected intravenously with 50 mg/kg of STZ. The STZ was freshly dissolved in citrate buffer (0.01 M, pH 4.5) and kept on ice prior to use. One week after STZ administration, the rats with FBG concentrations of over 150 mg/dl were considered to be diabetic and were used in the experiment.

### The oral glucose tolerance test (OGTT) in normal and STZ induced diabetic rats

After an overnight fasting, normal and diabetic rats were divided into five groups each with 6 rats in each group. Group I rats received 1 ml of distilled water only. Groups II - IV rats received various extracts of *Dregea volubilis* orally at 200 mg/kg respectively. Group V rats received 50 mg/kg Metformin. Glucose (3 g/kg) was administered orally to each rat 30 min later. Blood samples (0.5–0.6 ml) were collected from the tail vein in chilled heparinized tubes at −30, 0, 30, 90, 120 and 210 min for the estimation of blood glucose level. After centrifugation (2000 × g), plasma was removed and stored at −20°C. The plasma glucose concentrations were measured by the method of glucose oxidase - peroxides using Span diagnostic kits. After the pharmacological screening, it was found that among all the extracts, ethanolic extract showed a maximum decrease in FBG level. Hence, it was subjected to column chromatography and active principle was isolated.

### Isolation of active principle from ethanolic extract of *Dregea volubilis* leaf

Ethanolic extract of *Dregea volubilis* (10 g) was subjected to chromatography over a column of silica gel (60–120 mesh). The column was eluted successively with petroleum ether, petroleum ether – chloroform mixture, chloroform – methanol mixture in different proportion in the order of increasing polarity. Fractions with same Rf values on TLC were combined and evaporated to dryness under reduced pressure. The major active fraction (2.5 g) was obtained after elution with chloroform: methanol (70:30) and was further purified by chromatography over a column of silica gel (100–200) resulting in a yellow amorphous solid (2.0 g) after eluting with chloroform: methanol (75:25). An isolated fraction has been confirmed by GC-MS analysis which was given a trivial name Dv-1.

### Effect of DV-1 on FBG and lipid profile in diabetic rats

Normal and diabetic rats were divided into four groups with six rats in each group. Group I-normal rats received 1 ml of distilled water. Group II-diabetic rats received 1 ml of distilled water. Group III-diabetic rats received 100 mg/kg DV-1. Group IV-diabetic rats received 50 mg/kg Metformin. All the groups were treated orally for 21 days. At the end of the experimental period, the animals were fasted overnight for 8 hours and blood sample was taken from the retro orbital plexus under mild ether anaesthesia. Plasma was separated out and FBG level was measured by the method of glucose oxidase-peroxides using Span Diagnostic kits. Cholesterol level was determined by the enzymatic method (Masana et al. 2012), triglyceride by the enzymatic colorimetric method (Heber et al. 2013) and HDL by the phosphotungstate method (Tripathi et al. 2012) using span diagnostic kits in chemical under Methods.

### Identification of isolated fraction

Interpretation of GC-MS (Perkin-Elmer) was conducted using the database of National Institute Standard and Technology (NIST) having more than 62,000 patterns. The spectrum of the unknown components was compared with the spectrum of known components stored in the NIST library. The name, molecular weight, and structure of the components of the test materials were ascertained.

### Statistical analysis

Data are expressed as mean ± SEM. Statistical analysis was performed by one-way analysis of variance (ANOVA). The least significant difference test was used for mean comparisons and *p* < 0.05 was considered to be statistically significant.

## Results

### Acute toxicity study

Acute toxicity study showed that various extracts of *Dregea volubilis* did not produce any toxic symptoms when administered orally to rats. The lethal dose (LD_50_ value) was of 2000 mg/kg body weight.

### Effect of different leaf extracts of *Dregea volubilis* on FBG of normal and diabetic rats

Various extracts of *Dregea volubilis* (200 mg/kg) were evaluated in normal and diabetic rats along with the standard drug Metformin (50 mg/kg). In normal and diabetic rats, among all the extracts, ethanolic extract of *Dregea volubilis* reduced plasma glucose concentration significantly (*p* < 0.05) as like Metformin which was summarized in Tables 1 and 2.

**Table 1:** **Effect of different*Dregea volubilis*[Linn.] Benth leaf extracts on plasma glucose concentration in normal rats**

Treatments	Time (min) before and after glucose administration					
	−30	0	30	90	150	210
**Normal animals**	78.4 ± 3.1	81.6 ± 3.1	173.2 ± 1.6	136.4 ± 1.8	99.8 ± 5.8	88.2 ± 2.4
**Petroleum ether (200 mg/kg)**	75.7 ± 3.2	70.5 ± 4.9	175.6 ± 2.4	114.6 ± 3.4	106.7 ± 5.9	96.1 ± 1.4
**Ethyl acetate (200 mg/kg)**	79.4 ± 2.9	71.4 ± 4.7	142.3 ± 1.8	117.4 ± 2.4	102.4 ± 3.4	94.5 ± 4.8
**Ethanol (200 mg/kg)**	76.4 ± 2.6	72.4 ± 2.6	130.2 ± 1.9	99.1 ± 2.9	92.6 ± 2.3*	84.9 ± 3.6*
**Metformin (50 mg/kg)**	77.9 ± 3.1	81.8 ± 2.3	124.1 ± 2.5	96.4 ± 3.2	89.9 ± 3.1*	79.8 ± 2.8*

Data are expressed as mean ± SEM. *n* = 6 rats per group. *p < 0.05, compared to normal control group.

**Table 2:** **Effect of different*Dregea volubilis*[Linn.] Benth leaf extracts on plasma glucose concentration in diabetic rats**

Treatments	Time (min) before and after glucose administration					
	−30	0	30	90	150	210
**Normal animals**	174.8 ± 5.6	167.8 ± 3.1	234.1 ± 2.4	225.3 ± 1.9	188.3 ± 1.8	184.2 ± 2.4
**Petroleum ether (200 mg/kg)**	148.5 ± 3.2	146.7 ± 4.9	237.6 ± 2.4	221.6 ± 3.4	173.7 ± 5.9	168.1 ± 1.4
**Ethyl acetate (200 mg/kg)**	165.9 ± 2.9	153.1 ± 4.7	229.3 ± 1.8	195.4 ± 2.4	172.4 ± 3.4	164.5 ± 4.8
**Ethanol (200 mg/kg)**	170.4 ± 2.6	164.4 ± 2.6	224.2 ± 1.9	180.1 ± 2.9	142.6 ± 2.3*	96.9 ± 3.6*
**Metformin (50 mg/kg)**	171.9 ± 3.1	170.8 ± 2.3	226.1 ± 2.5	143.4 ± 3.2*	138.9 ± 3.1*	79.8 ± 2.8*

Data are expressed as mean ± SEM. *n* = 6 rats per group. *p < 0.05, compared to diabetic control group.

### Identification of isolated fraction and dose fixing

**Figure 1:**
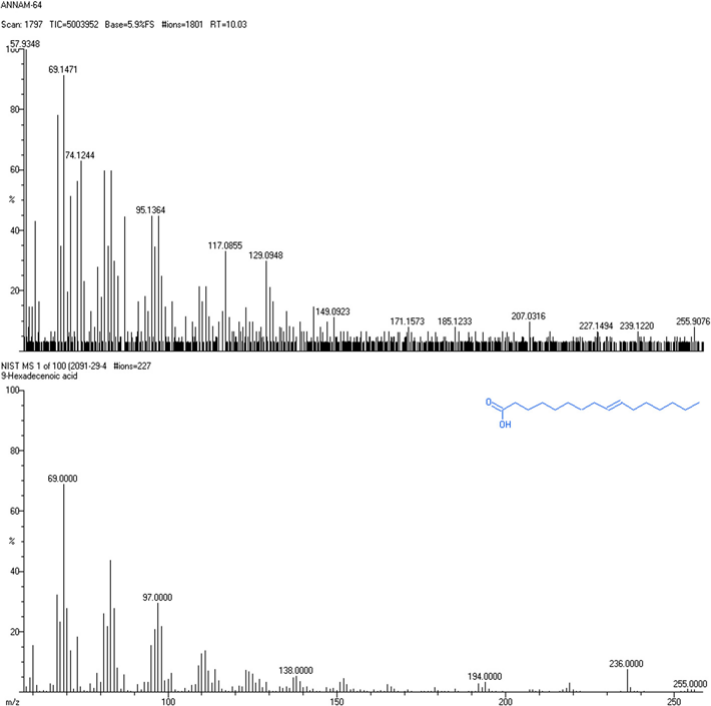
**GC-MS of isolated fraction Dv-1.**

Dv-1 was found to be the active principle of *Dregea volubilis* leaf. GC-MS confirmed the complete structure of Dv-1 and identified to be palmitic acid (Hexadecenoic acid) which was shown in Figure 1. Acute toxicity study revealed that Dv-1 did not produce any toxic symptoms when administered orally to rats. The lethal dose (LD_50_ value) was of 1000 mg/kg body weight.

### Effect of Dv-1 on FBG and the lipid profile of diabetic rats

Dv-1 produced significant (*p* < 0.05) reduction in FBG as like Metformin in diabetic rats summarized in Table 3. Additionally Dv-1 also caused significant (*p* < 0.05) reduction in the level of triglyceride and significant (*p* < 0.05) improvement in HDL. The effect was not much with cholesterol level.

**Table 3:** **Effect of oral administration of isolated fraction (DV-1) on plasma glucose concentration, cholesterol, triglyceride, and HDL for 21 days**

Treatment	Fasting plasma glucose (mg/dl)			Cholesterol (mg/dl)	Triglyceride (mg/dl)	HDL (mg/dl)
	0 day	10 day	21 day			
**Normal control**	64.8 ± 7.9	91.9 ± 3.2	89.3 ± 6.9	93.6 ± 4.3	71.6 ± 5.6	42.2 ± 1.9
**Diabetes alone**	249.7 ± 4.8	207.6 ± 8.5	195.7 ± 5.4	84.5 ± 3.4	110.6 ± 7.8	40.3 ± 2.1
**Diabetes + DV-1 (100 mg/kg)**	252.6 ± 4.1	186.3 ± 2.5*	161.2 ± 7.1*	87.6 ± 2.6	64.6 ± 7.5*	46.7 ± 2.3*
**Diabetes + Metformin (50 mg/kg)**	243.6 ± 2.8	178.8 ± 4.5*	156.8 ± 6.8*	84.3 ± 4.8	95.6 ± 5.6	47.6 ± 1.3*

Data are expressed as mean ± SEM. *n* = 6 rats per group. *p < 0.05, compared to diabetic control group.

## Discussion

Administration of streptozotocin caused rapid destruction of pancreatic cells in rats, which led to impaired glucose-stimulated insulin release and insulin resistance, both of which are marked feature of diabetes. The blood glucose-lowering effect of plant extracts is generally dependent upon the degree of pancreatic β cell destruction and useful in moderate streptozotocin-induced diabetes (Jianfeng et al. 2012). In general, an increase in blood glucose level is usually accompanied by an increase in plasma cholesterol, triglyceride, LDL levels and a decrease in HDL level as observed in diabetic patients (Henry et al. 2005). The marked hyperlipidemia (increase in the level of lipid in the body) that characterizes the diabetic state may be the consequence of the uninhibited actions of lipolytic hormones on fat depots (Saha et al. 2012). Among all the extracts tested, the ethanol extract produced significant reduction in the blood glucose level comparable to that of Metformin treatment. Isolated fraction Dv-1 was confirmed as hexadecenoic acid by gas chromatography, the compound peak being observed at 10.03 retention time. Further mass spectrum was performed to confirm structure of the compound. Hexadecenoic acid is a metabolite of the saturated fatty acid of palmitic acid that has been hydroxylated on its terminal (ώ) carbon. This ώ- hydroxylation of palmitic acid occurred by cytochrome P-450 in both plants and animals (Roman et al. 1993). Palmitic acid (Hexadecenoic acid) lowers blood glucose and alters the lipid profile (decreases TGL, cholesterol and improves HDL). Several investigators, however recognized that palmitic acid acutely stimulates glucose uptake via activated protein kinase and extracellular signal-related kinase (Jing et al. 2011) and produced great improvement of the altered lipid profile, it may also participates in the hypolipidemic activity by inactivating hepatic HMG-CoA reductase a key enzyme, in cholesterol synthesis. The improvements in the lipid profile in diabetic animals after treatment with palmitic acid (Hexadecenoic acid) could be beneficial in preventing diabetic complications. With the research carried out, hexadecenoic acid from the ethanolic extract of *Dregea volubilis* leaf has shown significant reduction in blood glucose and alteration in the lipid profile in diabetic rats.
